# Current Evidence on the Use of Calcitonin Gene-Related Peptide-Targeting Therapies in Children and Adolescents

**DOI:** 10.1007/s11916-026-01567-4

**Published:** 2026-10-14

**Authors:** Lauren Shin, Ryan Moon, Amy Gelfand

**Affiliations:** Benioff Children‘s Hospital—Child & Adolescent Headache Program, University of California—San Francisco, San Francisco, California USA

**Keywords:** Migraine, Calcitonin-gene-related peptide (CGRP), Headache, Adolescent, Pediatric

## Abstract

**Purpose of Review:**

This is a narrative review to summarize current evidence on use of therapies targeting calcitonin gene-related peptide (CGRP) for children and adolescents with headache disorders.

**Recent Findings:**

The CGRP-targeting therapies have been used in adults for prevention of migraine since 2018. There is one randomized controlled trial (RCT) published on fremanezumab in children age 6–17 years old which showed favorable outcome as a preventive treatment for episodic migraine. Several real-world evidence (RWE) studies report on use of CGRP-targeting therapies in patients younger than 18 years old. Several clinical trials are pending for other CGRP-targeting therapies.

**Conclusion:**

There is a small number of RWE studies and one RCT study that shows that CGRP-targeting therapies have potential to be beneficial and safe to use in children and adolescents with headache disorders. Future clinical trials, once completed, will further shed light on efficacy and safety of CGRP-targeting therapies.

## Introduction

Headache disorders are some of the most common neurologic disorders seen in children and adolescents. An estimated 1 in 10 children and adolescents has migraine [1]. Other chronic headache disorders experienced by children and adolescents include persistent post-traumatic headache (PPTH) and new daily persistent headache (NDPH) [2, 3]. Chronic headache disorders can lead to absences from school, missed extracurriculars, general disability in day-to-day life activities, and overall poorer quality of life in the pediatric population [4–6].

One of the revolutionary advancements in migraine therapeutics has been therapies targeting calcitonin gene-related peptide (CGRP). CGRP has been implicated in migraine pathophysiology since 1980s [7–12]. CGRP-targeting therapies became first commercially available in the U.S. in 2018. The first CGRP inhibitor approved by the Food and Drug Administration (FDA) in adults was erenumab, a monoclonal antibody to the canonical CGRP receptor for use in migraine prevention. In subsequent years, other CGRP inhibitors have shown efficacy and tolerability leading to FDA indication for use in migraine treatment for adults [13–23]. Galcanezumab has an additional indication for prevention of episodic cluster headaches [24], demonstrating that CGRP inhibitors may have therapeutic benefit beyond migraine.

In the pediatric population, fremanezumab was recently approved by FDA for treatment of episodic migraine in age 6–17 years old following its publication of phase 3 study demonstrating efficacy and safety [25]. Other randomized controlled trials (RCT) in pediatric populations are currently ongoing [26]. These trials are projected to be completed in 2026–2032. Based on the favorable outcomes in adult trials and clinical experience, CGRP inhibitors have been used off-label in the pediatric setting for treatment of chronic headache disorders [27–29]. The aim of this review is to summarize the currently available evidence regarding the use of CGRP-targeting therapies in children and adolescents.

## CGRP in Headache Disorders

Calcitonin gene-related peptide has been a therapeutic target in migraine research for several decades. CGRP is a vasodilatory neuropeptide released in the trigeminovascular complex associated with migraine pathogenesis [7–9, 12]. CGRP and its receptors are expressed in various areas within central and peripheral nervous system as well as in other systems in the body [30, 31]. There has been much clinical evidence that CGRP is linked to migraine. In adults with migraine, plasma CGRP levels are increased compared to those who do not have migraine and are persistently elevated in those with chronic migraine [32, 33]. This has also been seen in the pediatric population as well [34]. Intravenous administration of CGRP in patients with migraine induced migraine attacks suggesting a causal role of CGRP [35].

There is some evidence that CGRP plays a role in other chronic headache disorders as well. In cluster headache (CH), CGRP can be released during spontaneous and induced attacks [36, 37]. CGRP has also been linked to post-traumatic headache where elevation of CGRP level is seen following multiple head injuries in rat models of post-traumatic headaches [38]. In turn, an early and continuous blockade of CGRP in mice models of acute and persistent post-traumatic headache was associated with decreased likelihood of developing allodynia [39].

There are two classes of CGRP-targeting therapies in the U.S. market – anti-CGRP monoclonal antibodies (mAbs) and small molecule CGRP receptor antagonists called gepants.

## Current Available CGRP-Targeting Therapies

### Anti-CGRP Monoclonal Antibodies

There are four anti-CGRP mAbs available in the U.S for prevention of migraine in adults that are FDA approved following multiple RCTs showing efficacy and tolerability – erenumab [15, 17, 40–42], fremanezumab [14, 18, 43, 44], galcanezumab [19, 45–47], and eptinezumab [16, 20, 48, 49]. The anti-CGRP mAbs may bind to the CGRP receptor (erenumab) or the CGRP ligand (fremanezumab, galcanezumab, eptinezumab). Erenumab, fremanezumab and galcanezumab are available as subcutaneous injection administered every 28 days. Eptinezumab is an intravenous formulation administered every 3 months. The RCTs generally report mild or nonserious adverse effects. The most common adverse events for erenumab, fremanezumab, and galcanezumab is an injection site reaction (e.g. pain, erythema, pruritus) [17–19]. Constipation is also seen with erenumab. The most common adverse event with eptinezumab is nasopharyngitis. Hypersensitivity reactions (pruritus, flushing, hypersensitivity that occurred on the day of dosing) have also been reported in the study participants receiving eptinezumab. In addition, new-onset hypertension or worsening of pre-existing hypertension as well as Raynaud’s phenomenon with use of anti-CGRP mAbs have been reported in the post-marketing setting [50–52]. Galcanezumab RCTs showed efficacy in episodic cluster headache in adults but did not for chronic cluster headaches, hence the FDA indication is only for episodic cluster headaches [24, 53].

### Gepants

There are four gepants available in the U.S for treatment of migraine in adults – rimegepant [22, 54–56], atogepant [21, 57–59], ubrogepant [23, 60, 61], and zavegepant [62, 63]. Gepants bind to the CGRP receptor to block CGRP from binding to its receptors. Rimegepant has indication for both prevention and acute treatment for migraine in adults. Atogepant is used for prevention of migraine. Ubrogepant and zavegepant are used as acute treatments for migraine. Of note, ubrogepant may have a “prophylactic” role in mitigating a migraine attack during the prodromal phase [64]. The RCTs of these medications also report generally mild and nonserious adverse effects. Adverse effects of rimegepant include nausea, abdominal pain, and dyspepsia. Adverse effects of atogepant include fatigue, nausea, and constipation. Adverse effects of ubrogepant include nausea and somnolence. Adverse effects of zavegepant include taste disorder, nausea, vomiting, and nasal discomfort.

## CGRP-Targeting Therapies in the Pediatric Population

Although all anti-CGRP mAbs and gepants have had favorable RCTs for treatment of migraine in adults, at the time of this review, only fremanezumab has published results of its RCT for use in children aged 6 to 17 years old. Nonetheless, others have been used off label in the pediatric population, particularly in adolescents. We’ll highlight results from available studies for each class of medication that have been used in children and/or adolescents. Summary of the available evidence in children and/or adolescents is in Table 1.

**Table 1:** Summary of Current Available Evidence for Use of CGRP-Targeted Therapies in Children and/or Adolescents

First Author and Year Published	Study Type	Sample size (*n*)	Age	Medications Studied	Outcome Highlights	Safety Notes
Anti-CGRP Monoclonal Antibodies						
Hershey et al., 2026	Randomized double-blind placebo controlled trial	234 total; fremanezumab, *n* = 123; placebo, *n* = 111	Mean age of 13.3 years in the fremanezumab group (range 6–17) 6–11 years old, *n* = 32; 12–17 years old, *n* = 91	Fremanezumab for prevention of episodic migraine	• Mean change of MMD from baseline in 3-month follow up period: -2.5 [95% CI, − 3.2 to –1.7] vs. -1.4 days [95% CI, − 2.2 to − 0.7]; (*p* = 0.02) in the fremanezumab group and placebo group respectively • Mean change of days per month with headache of at least moderate severity: − 2.6 days [95% CI, − 3.4 to –1.8] vs. − 1.5 days [95% CI, − 2.3 to − 0.7]; (*p* = 0.02) • Reduction of 50% or more in MMD: 47.2% vs. 27% in the fremanezumab and placebo group respectively (*P* = 0.002). • Mean change from baseline in average number of days per month in which acute head‑ache medication was used: − 2.1 days (95% CI, − 2.6 to − 1.5) with fremanezumab and − 1.0 days (95% CI, − 1.6 to − 0.4) with placebo (*p* = 0.002)	• Most AEs were nonserious and mild to moderate in severity • 68 of 123 participants (55.3%) receiving fremanezumab and 55 of 112 (49.1%) receiving placebo had at least one AE • The most common AE was injection-site erythema − 9.8% (12 of 123) in the fremanezumab group
Hershey et al., 2026	Randomized double-blind placebo controlled trial	289 total; fremanezumab, *n* = 149; placebo, *n* = 140	Not available; recruited age 6–17 years old	Fremanezumab for prevention of chronic migraine	• Abstract only • No difference in change from baseline in MMD between fremanezumab versus placebo (–3.8 vs. − 3.7; *p* = 0.8484), with reductions in MMD observed for both treatment groups	• % of participants reporting at least 1 AE: 58% vs. 54% in fremanezumab vs. placebo groups
Cohen-Barak et al., 2021	Dose selection for Phase 3 based on open-label phase 1 study	15	Mean age was 9.3 years (range 6–11)	Fremanezumab	• Participants received a single dose of 75 mg of fremanezumab SC • Primary end points from phase 1 were mainly pharmacokinetics data such as maximum plasma drug concentration • In dose selection, 120 mg SC deemed appropriate for weight < 45 kg and 225 mg SC for weight > 45 kg	• There were no serious adverse events reported. • 7 (47%) patients reported at least one AE, the majority of which were of mild severity (6 [40%] patients) • 5 patients (33%) reported injection site erythema.
Hershey et al., 2025	Open-label PK clinical trial	28 total	Mean age of 12.3 years (range 6–17) 6–11 years old, *n* = 12; 12–17 years old, *n* = 16	Eptinezumab	• Doses of 150 mg and 300 mg in each age group resulted in similar mean plasma concentration-time curves after a single injection • Change in PedMIDAS score between pre-treatment and post-treatment was − 22 in children and − 17 in adolescents (exploratory analysis)	• 57% (16/28) experienced one or more TEAE. • The most common TEAE: orthostatic hypotension in three adolescents. • No serious AE or severe TEAE were reported
Greene et al., 2021	Retrospective chart review	112	Mean age of 15.9 years at time of first dose (range 10.3–17.8)	Erenumab, fremanezumab, galcanezumab	• At the first follow-up visit (mean time 2.7 months), 29.5% of patients indicated “significant” benefit, 42.0% indicated “some” benefit, 24.1% indicated no benefit, and 4.5% indicated their worsening of their headaches. • At the second follow-up visit (mean time 4.6 months), 30.1% of patients indicated significant benefit, 39.7% indicated some benefit, 23.3% indicated no benefit, and 6.8% indicated their worsening of their headaches. • Improvement in function reported – 61.7% (58/94) reported at least some improvement in function at the first follow up and 53.7% (36/67) reported at least some improvement in function at the second follow up.	• 32.1% (36/112) reported at least one SE • The most common SE: injection site reactions (17% (19/112)) • Other reported SE: 8% constipation (9/112), fatigue 7.1% (8/112), 4.5% dizziness (5/112), 3.6% nausea and worsening of headache (4/112).
Bandatmakur et al., 2024	Retrospective chart review	23; Of note, 14 patients used anti-CGRP agents for prevention only; 9 patients used them for both prevention and as a rescue	9 patients were in the age range of 12–15 years, 12 in the age range of 16–18 years, and 2 were > 18 years.	Erenumab, fremanezumab, galcanezumab, ubrogepant, rimegepant, and/or atogepant;	• 21 (91.3%) patients reported a reduction in duration and intensity of migraines • 82.6% reported improvements in other bothersome symptoms • After one month, 82.6% reported a reduction in headache days • After three months, 87% reported a reduction in headache days • ~ 40% of patients experiencing more than a 50% reduction at each time point (1 month and 3 months) • 69.6% of patients expressed strong satisfaction with their treatment outcomes	• 22 (95.7%) patients reported no side effects • 1 patient reported “few” side effects but manuscript does not describe them
Zhao et al., 2020	Case series	6	Age range of 15–18 years	Erenumab	• Reported > 50% reduction in mean monthly headache days in 2 patients, subjective improvement in 1 patient, and 3 had no response to erenumab.	• 1 patient reported constipation
Katsuki et al., 2023	Case series	10	Mean age was 15.9 years (range of 15–17)	Erenumab, fremanezumab, galcanezumab	• Median HIT-6 score went from 63 to 44 after 3 months. • Median MHD before and 3 months after treatment was 5.5 (1–29) and 1.5 (1–30), respectively but no statistical significance (*p* = 0.241) • Median AMD was 5.5 (1–30) and 1 (0–30), respectively but no statistical significance (*p* = 0.284)	No reported side effects
Zorrilla et al., 2023	Retrospective chart review	11	Mean age was 17 years range 14–20)	Eptinezumab	• Nine out of 11 patients reported some improvement after their infusion/s of eptinezumab. • Two stopped having daily headaches and one stopped having a continuous headache • Four reported reduction in headache intensity, one reported reduction in nausea and vomiting, and one reported improved function.	• 1 patient reported nausea with second infusion • 1 patient reported worsening of baseline headache after first infusion
Na et al., 2025	Retrospective chart review	10	Median age at first dose was 14 years (range 12–16)	Galcanezumab, fremanezumab, or atogepant along with lifestyle interventions	• PedMIDAS score was decreased from a median of 58.5 at baseline to 48.0 at 12 months. Monthly migraine frequency decreased from a median of 26.5 days per month at baseline to 14.0 days at 12 months.	• No serious SE reported • 5 patients reported injection site pain and redness, 1 reported myalgia, and 2 reported dizziness.
Akbar et al., 2025	Case series	8	Median age was 17.2 years (range 15.1–17.8)	Galcanezumab or erenumab	• Median HIT-6 had decreased from 76 to 36 after 6 months • Median PedMIDAS score decreased from 40 to 5 after 6 months • All reported positive response	• 1 patient reported constipation and fatigue with galcanezumab
Gepants						
Shin et al., 2026	Retrospective chart review	71 total Rimegepant (*n* = 55) Atogepant (*n* = 26) Both rimegepant and atogepant (*n* = 10)	Median age of the Rimegepant group was 16.0 years (range 14.4–17.1). Median age of the Atogepant group was 16.6 years (range 15.2–17.1).	Preventative atogepant and preventative rimegepant	• 58% (32/55) of the rimegepant group and 42% (11/26) of the atogepant group reported at least some benefit • The number of patients reporting “significant functional limitation” decreased in the rimegepant group between pretreatment and posttreatment − 22/55 (40%) to 7/54 (13%); and in the atogepant group from 13/26 (50%) to 5/26 (19%)	• SE were mild and nonserious • In the rimegepant group,1 patient reported constipation, 1 reported nausea, 1 reported abdominal pain and fatigue, and 1 reported transient worsening of headache. • In the atogepant group, 2 patients reported nausea, 2 reported fatigue, 1 reported constipation, 1 reported depression, and 1 felt “cold all over.”
Tourlas et al., 2026	Retrospective chart review	12	Mean age was 15.3 (range 13–17)	Rimegepant as acute treatment only	• After at least one dose of rimegepant, clinical benefit reported in 83% (10/12) of the patients within 2 h o 3 patients reported pain freedom o 7 patients reported partial pain relief	• No reported side effect
Onifade et al., 2023	Retrospective chart review	59	Not available; <18 years old	Rimegepant or ubrogepant as acute treatments	• Abstract only • 32% of the patients reported full pain relief “sometimes” • 17% of the patients reported full pain relief “always” • 36% of the patients reported partial pain relief “sometimes” • 32% of the patients reported partial pain relief “always”	• 89% did not have SE • Reported side effects included nausea/vomiting, fatigue, dizziness, worsening headache, and anxiety

AE = Adverse event

AMD = Monthly acute medication intake days

CI = Confidence interval

MMD = Migraine days per month

HIT-6 = Headache Impact Test-6

MHD = Monthly headache days

PedMIDAS = Pediatric Migraine Disability Assessment

PK = Pharmacokinetics

SE = Side effect

SC = Subcutaneous

TEAE = Treatment emergent adverse event

### Anti-CGRP Monoclonal Antibodies

#### Fremanezumab

Fremanezumab was approved in August 2025 by FDA for use in age 6–17 years (who weigh at least 45 kg) for prevention of episodic migraine (EM) [18]. The labeled dose is 225 mg monthly. Of note, there is an additional dosing schedule, which is 675 mg every 3 months for adults. However, this dosing is not approved in the pediatric population. The results of the phase 3 double-blind RCT in children and adolescents were published in January 2026^65^. A total of 234 participants were included in the study analysis. Of the 123 participants in the fremanezumab group, 36 received the 120 mg dose and 87 received the 225 mg dose based on the weight cutoff of 45 kg. The efficacy reported in this trial is comparable to the results from the adult study. There was reduction in moderate or severe headache days (mean change from baseline, -2.6 days vs. -1.5 day with placebo; difference, 1.1; *p* = 0.02), migraine days (mean change from baseline, -2.5 days vs. -1.4 with placebo; difference, 1.1; *p* = 0.02), and usage of acute medication in the fremanezumab group compared to the place group (mean difference between groups, -1.1 days *p* = 0.002). There was approximately 1 day difference in reduction of headache and migraine days between active and placebo. In adults with episodic migraine, the reduction of migraine days per month was 1.5 days greater with fremanezumab than with placebo [14]. The percentage of participants who had a reduction of ≥ 50% in the number of migraine days per month was 47.2% with fremanezumab and 27.0% with placebo (*p* = 0.002). Again, this result was similar to the adult trial as well (47.7% and 27.9% respectively for the fremanezumab and placebo groups). There was not a statistically significant difference in change in Pediatric Migraine Disability Assessment (PedMIDAS) scores between the groups.

As for safety, 55% (68/123) of the fremanezumab group had at least one adverse event reported compared to 49% (55/112) of the placebo group, however most adverse events were mild. The most common adverse event with fremanezumab was injection-site erythema (9.8% of participants vs. 5.4% with placebo). Serious events were reported by two participants in the fremanezumab group (hepatitis secondary to infectious mononucleosis and migraine exacerbation leading to hospitalization) and three participants in the placebo group (thrombocytopenia, hemiparesis, migraine). No deaths occurred. A mild safety profile was reported in the phase 1 pharmacokinetic study of fremanezumab in children aged 6–17 years old [65]. In this study, fifteen pediatric patients were enrolled an open-label study. Seven (47%) patients reported at least one adverse event. The most common adverse event was injection site erythema (5/15, 33%). No death, serious adverse event, or treatment-emergent adverse events (TEAE) were reported.

The use of fremanezumab in children and adolescents with chronic migraine (CM) is available only as an abstract at the time of this review [66]. In the CM study, 289 participants were randomized between fremanezumab and placebo. There was no difference in change from baseline in mean migraine days between fremanezumab versus placebo in the CM study (–3.8 vs. − 3.7; *p* = 0.8484). There was similar proportion of participants reporting at least one adverse event – 58% of the fremanezumab group vs. 54% of the placebo group. The full study results are yet to be published.

#### Eptinezumab

The pharmacokinetics study of eptinezumab in children and adolescents reported on safety outcomes and its effect on level of functional disability evidenced by PedMIDAS [67]. Twenty-eight participants were included, 12 of whom were aged 6–11 years and the rest were aged 12–17 years. Doses of 150 mg and 300 mg in each respective group resulted in similar mean plasma concentration-time curves after single injection. There was no serious adverse event or severe TEAE. The most common TEAE was orthostatic hypotension reported in three adolescents, which resolved within the same day. There was reduction in the mean PedMIDAS scores in both pediatric and adolescent groups at the week 12 follow up.

#### Monoclonal Antibodies in Real-World Practice

Other studies reporting anti-CGRP mAb usage in children and adolescents have been real-world evidence (RWE) studies where anti-CGRP mAbs are prescribed off label for treatment of migraine or other headache disorders.

The largest RWE study is a multi-center retrospective cohort study published in 2021 by Greene et al., which reviewed the use of anti-CGRP mAbs (erenumab, galcanezumab, or fremanezumab) in patients under 18 years of age in three tertiary pediatric headache centers [28]. A total of 112 patients were included in this study where they received at least one dose of any anti-CGRP mAbs for treatment of CM, NDPH, or PPTH (83.9% had CM). In this study, 71.5% (80/112) of the patients reported at least some benefits at the first follow up and 69.8% (51/73) reported at least some benefit at the second follow up. There was reduction of headache days per month by 2 days (95% CI: −0.8 to − 3.2). There was improvement in function reported – 61.7% (58/94) reported at least some improvement in function at the first follow up and 53.7% (36/67) reported at least some improvement in function at the second follow up. The most common side effects were injection site reactions in 17.0% (19/112) and constipation in 8.0% (9/112) of patients. Five patients (4.5%) discontinued due to side effects. It is notable that the patients included in this study had high headache burden evidenced by the mean number of headache days per month (26.9, SD 6.1), presence of continuous headache in majority of participants (67.6%), and the mean number of prior preventive trials of 9.5 (SD 5.1).

A smaller retrospective study published in 2024 by Bandatmakur et al. reports on the use of anti-CGRP agents in adolescents aged 12 to 21 years old with chronic headache disorders [68]. Twenty-three patients were included in the study, and of these, two patients were over the age of 18. Most of the patients had CM as the primary diagnosis (78%, 18/23) – others had EM (*n* = 3), NDPH (*n* = 1), and PPTH (*n* = 1). Most of the patients (60.9%) used anti-CGRP agents primarily for prevention, and 39.1% used them for both acute use and prevention. The majority (65.2%, 15/23) only used anti-CGRP mAb, some used (13%, 3/23) only gepants as their primary therapy and some (21.7%, 5/23) used both CGRP-mAbs and gepants. The study reports outcome from the whole group only and does not separate them into subgroups. Twenty-one patients (91%) reported at least some reduction in duration and intensity of migraine attacks. There was also reduction in bothersome symptoms, emergency department visits, and use of rescue medication in a week. Nine patients (39%) saw ≥ 50% reduction in number of headache days per month. There was a high patient satisfaction, with 69% (16/23) reporting strong satisfaction. One patient reported side effects (unspecified in the manuscript) while others had none.

One of the earliest RWE study was a case series by Zhao et al. in 2020 which included six adolescents aged 15 to 18 years with EM or CM treated with erenumab [69]. At baseline, the mean monthly migraine days (MMD) was 22.3 days and four of them had been unresponsive to more than three prior preventive treatments. There was favorable response seen in two patients where MMD reduced from 30 days to 10 days after one month of treatment. There was no response seen in three patients, and one patient reported subjective improvement. Constipation was reported in one patient.

Katsuki et al. reported on usage of anti-CGRP mAbs (fremanezumab, galcanezumab, or erenumab) in ten Japanese adolescents aged 15–17 years in Japan with migraine [70]. There was significant reduction in Headache Impact Test-6 (HIT-6) score (*p* = 0.008) – from 63 to 44 after 3 months. Although statistically significant reduction was not seen in median headache days per month or acute medication intake days, there was numeric reduction in both measures.

Zorrilla et al. reported findings from use of eptinezumab in eleven adolescents who had daily headaches (9 patients with CM and 2 patients with NDPH) [29]. After first infusion of 100 mg, two patients stopped having daily headaches, one patient stopped having a continuous headache, and four patients reported reduction in the headache intensity. With subsequent infusions, some reported benefit. Three patients opted to receive 300 mg when they felt the effect from 100 mg had “plateaued.” Of these three, one patient had a reduction in headache days per month (12 to 8). One patient reported a reduction in headache pain intensity (9 to 5 out of 10). The third patient reported no benefit in intensity or frequency. One patient reported nausea with second infusion and one reported worsening of baseline headache after first infusion.

A study by Na et al. included ten Korean adolescents who received anti-CGRP mAbs or a gepant for prevention along with lifestyle interventions [71]. Migraine frequency decreased from a median of 26.5 to 14 days per month (*p* < 0.001). PedMIDAS scores decreased from 58.5 to 48.0 (*p* = 0.037). Use of acute treatment was reduced from 14 to 5 days per month (*p* < 0.001). Side effects included site pain and dizziness.

Lastly, a case series by Akbar et al. from 2026 included eight patients under 18 years old who had chronic daily headaches (with primary diagnoses of CM, PPTH, or NDPH) and received anti-CGRP mAbs [72]. All patients reported positive response and the median HIT-6 and PedMIDAS scores decreased from 76 to 40 to 36 and 5 respectively. One patient reported constipation and fatigue with galcanezumab.

### Gepants

#### Gepants as Preventive Treatments

At the time of this review, gepants do not have any RCT or open-label trials published in the pediatric population. The largest study to date is a multi-site retrospective study published in 2025 on the use of gepants as preventive treatments in children and adolescents under 18 years old [27]. Of the 71 patients included in the study, 26 used atogepant, 55 used rimegepant, and 10 used both. The primary diagnoses included CM, NDPH, and PPTH. In the rimegepant group, 58% (32/55) reported at least some benefit. In the atogepant group, 43% (11/26) reported at least some benefit. Side effects were mild and nonserious. In the rimegepant group, 4 patients reported side effects including constipation, nausea, abdominal pain, fatigue, and transient worsening of headache (*n* = 1 for each). In the atogepant group, 7 patients reported side effects which included nausea (2/26), fatigue (2/26), constipation (1/26), depressed mood (1/26), and feeling cold (1/26). It is notable that patients included in this study also had a high headache burden evidenced by the number of headache days per month (median of 30), number of prior preventive trials (median of 6 or 7 in each group), and a large proportion of patients with severe functional limitations (40% in the rimegepant group and 50% in the atogepant group).

#### Gepants as Acute Treatments

Most recently, a small retrospective study was published on use of rimegepant as an acute treatment in adolescents with migraine [73]. Twelve patients with episodic, chronic, or hemiplegic migraine (HM) were included. Ten patients reported positive outcome where three reported pain freedom and the others reported pain improvement within 2 h of using rimegepant. The mean number of previously tried acute treatments was 3.9 (SD +/- 2.4). No side effects were reported. Weight, height, blood pressures were checked pre- and post-treatment in this study, and there was no significant difference.

There is one abstract presented in 2023 on the use of gepants as acute treatments [74]. In this study, 59 patients under the age of 18 years old were prescribed and had used rimegepant or ubrogepant as acute treatments. Of 59 patients, 49% of the patients reported full pain relief “sometimes” (32%) or “always” (17%). In the same population, 68% reported frequency of partial pain relief as “always” (32%) or “sometimes” (36%). Most of the patients (89%) did not have side effects. Reported side effects included nausea/vomiting, fatigue, dizziness, worsening headache, and anxiety. We are not aware of any reports on the use of zavegepant in the pediatric population, though a clinical trial is currently underway (NCT06995729, clinicaltrials.gov, accessed March 27th, 2026). 

## Discussion

There is a paucity of evidence-based pharmacologic treatments for children and adolescents with chronic headache disorders especially in prophylaxis [75, 76]. Topiramate and fremanezumab are the only FDA-approved migraine prophylactic treatments approved in ages 12–17 and 6–17 respectively as of 2026. One of the challenges in developing a pharmacologic treatment in the pediatric population has been due to a high response rate to a placebo when a treatment undergoes a clinical trial [77–79]. In the CHAMP trial, there was no significant difference between amitriptyline, topiramate, and placebo groups when used for migraine prevention as all groups saw clinical improvement [77]. The preliminary results from the fremanezumab RCT for treatment of CM seem discouraging at first that there was not a significant difference between the fremanezumab group and the placebo group, however both groups did see clinical improvement. The fremanezumab RCT for EM and the RWE studies otherwise provide a hopeful glimpse into the potential therapeutic benefit of CGRP-targeting therapies in children and/or adolescents. It is notable that the retrospective RWE studies generally include patients with high headache burden whose disease had been refractory to several medications prior to the initiation of CGRP-targeting therapies. This may introduce selection bias where studies are reporting results from a particularly refractory population. On the other hand, these patients often are excluded from RCTs so it is encouraging that even those with refractory disease may respond positively to CGRP-targeting therapies. Moreover, the multiple preventive trials prior to use of an CGRP inhibitor suggest that the reported improvement may not merely be from chance or placebo effect. RWE studies should be interpreted cautiously as they all carry several limitations including small sample size, lack of control groups, and missing data. Some outcome measures include subjective reporting of improvement which may not reflect quantifiable benefit.

Studies generally show mild and nonserious adverse effects in both children and adults, however, it should be noted that long-term effects from CGRP-targeting therapies are not yet fully known. In post-marketing studies in adults, an increased risk of hypertension and Raynaud’s phenomenon have been reported [50–52]. There is also little research done on safety when using a combination of anti-CGRP agents. A small number of studies in adults shows general safety in combining anti-CGRP mAb and gepants but more studies are needed [80–82].

Recommendations from professional societies of headache medicine have been supportive of using CGRP targeting therapies in migraine. The position statement from American Headache Society (AHS) in 2024 states that CGRP targeting therapies should be considered one of the first-line therapies for migraine prevention and outlines recommendations on their use [83]. In 2022, European Headache Federation updated its guideline on using CGRP mAbs for migraine prevention that they be considered part of the first line treatment options based on moderate to high level of evidence from the clinical trials [84]. Given the unknowns in the pediatric population, there is not a clear guideline for this population yet. In 2018, an opinion piece was published by authors within the Pediatric Headache Special Interest Group of AHS regarding recommendations on the use of anti-CGRP-mAbs in children and adolescents [85]. With more clinical experience since its publication, some of the recommendations such as monitoring parameters may be less relevant. Once more evidence is available, there hopefully will be more guidance on usage of these medications in the pediatric population.

## Conclusion

At the time of this review, fremanezumab is the only CGRP-targeting therapy that has published strong evidence for use in children and adolescents with episodic migraine. Other CGRP inhibitors are undergoing clinical trials, which will be completed in the next few years. Given the limited number of evidence-based pharmacologic treatments in pediatric headache disorders, these studies will be invaluable. The RWE studies support favorable outcome and tolerability. However, given the inherent limitations of retrospective studies, the results of these studies are to be interpreted with caution. In the meantime, the use of CGRP inhibitors in children and adolescents may be reasonable in some, especially those with migraine.

## Key Reference

- Hershey, A. D. et al. Fremanezumab in Children and Adolescents with Episodic Migraine. N Engl J Med 394, 243-252, doi:10.1056/NEJMoa2504546 (2026).
  - The first published randomized controlled trial of an anti-CGRP monoclonal antibodies in children and adolescents.
- Evers, S. CGRP in Childhood and Adolescence Migraine: (Patho)physiological and Clinical Aspects. Curr Pain Headache Rep 26, 475-480, doi:10.1007/s11916-022-01047-5 (2022)
  - A review of CGRP’s roles and metabolism in children and mechanism in treatent of migraine in children.
- Gelfand, A. A. & Szperka, C. L. What's in the pipeline for pediatric headache treatment? Headache 66, 548-550, doi:10.1111/head.70037 (2026).
  - A brief overview of a number of pending clinical trials of migraine treatments in the pediatric population.

## Data Availability

No datasets were generated or analysed during the current study.

## Declarations

### Humans and Animals Rights

This article does not include any studies with human or animal subjects performed by any of the authors.
